# Drag Voltages in a Superconductor/Insulator/Ferromagnet Trilayer

**DOI:** 10.3390/ma14247575

**Published:** 2021-12-09

**Authors:** Paola Romano, Albino Polcari, Carla Cirillo, Carmine Attanasio

**Affiliations:** 1 Science and Technology Department, University of Sannio, I-82100 Benevento, Italy; a.polcari@libero.it; 2 CNR-SPIN, c/o University of Salerno, I-84084 Fisciano, Italy; carla.cirillo@spin.cnr.it (C.C.); cattanasio@unisa.it (C.A.); 3 Liceo Statale “F. De Sanctis”, I-84133 Salerno, Italy; 4 Physics Department “E.R. Caianiello”, University of Salerno, I-84084 Fisciano, Italy

**Keywords:** trilayers, drag voltages, transport measurements

## Abstract

The interaction between two spatially separated systems is of strong interest in order to study a wide class of unconventional effects at cryogenic temperatures. Here we report on drag transverse voltage effects in multilayered systems containing superconducting and ferromagnetic materials. The sample under test is a conventional superconductor/insulator/ferromagnet (S/I/F) trilayer in a cross configuration. S/F as well as S/N (here N stands for normal metal) bilayers in the same geometry are also analyzed for comparison. Current–voltage (I–V) characteristics measured at T = 4.2 K in the presence of a perpendicular magnetic field show strong peculiarities related to the interaction between the layers. The results are interpreted in terms of interaction effects between the layers.

## 1. Introduction

**Figure 1:**
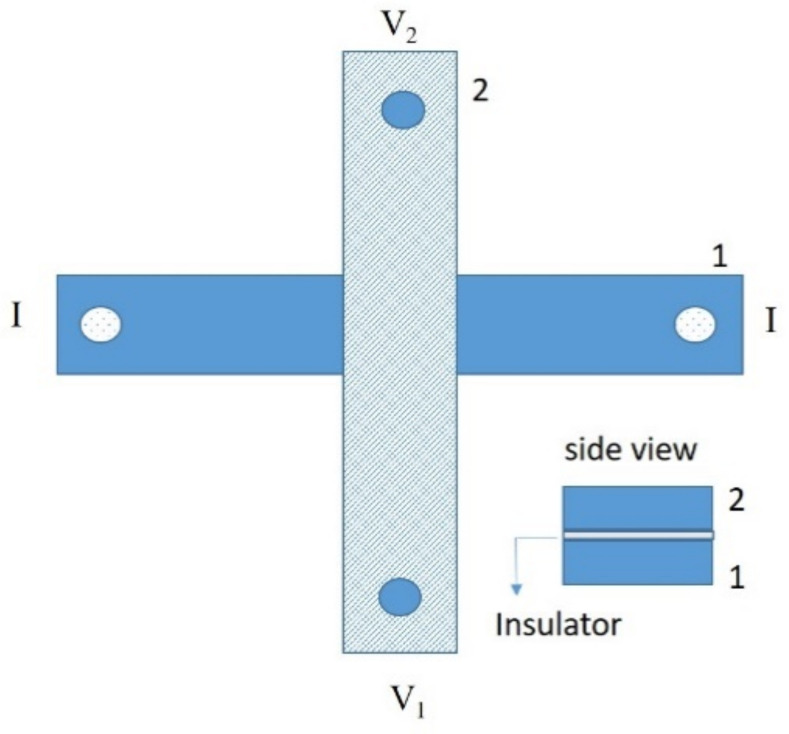
Schematic of the transport measurement geometry in cross configuration (planar and side view). I is the bias current and V = V_2_−V_1_ is the voltage drop.

Among all the systems in which the study of the electric transport properties is a powerful tool to investigate fundamental phenomena, double-layer structures consisting of two parallel quantum wells separated by a potential barrier are an important class of systems because of potential applications as electronic devices at the nanoscale [1,2,3]. Each layer hosts a quasi-two-dimensional electron gas and electrons interact across the barrier via the Coulomb interaction. When an electric current is driven in one of the layers, the Coulomb interaction causes a charge accumulation in the other layer, in which no current flows. This phenomenon is called Coulomb drag [1,2,3] and it has been largely hypothesized in two-dimensional electron systems in several configurations [4,5,6,7,8,9] and also observed in N/I/S structures based on low-T_c_ superconductors [10,11] as well as theoretically analyzed in spin-valve systems [12]. At the same time, S/F heterostructures have recently received great attention in the scientific community. This attention is not only for the basic investigation of peculiar phenomena occurring in these systems but also for application purposes especially in the field of spintronics [13,14,15], as in the case of S/F/S Josephson junctions [16,17,18] or heterostructures devices such as F/S/F spin valves [19]. Transverse voltage effects induced by Coulomb drag between high-temperature superconductors and a ferromagnetic barrier in a cross configuration has also been recently observed [20]. Typically, a conventional four terminal configuration is used to test the electrical response of a sample [21]. In our case, being the sample made by two layers, two contacts are on the electrode 1 and the other two contacts are on the electrode 2. The four contacts are not in line but arranged in a cross configuration, as shown in Figure 1. The two electrodes are separated by means of an insulating barrier; in this way we can measure possible induced voltage effects in the unbiased layer.

Drag measurements allow to study fundamental properties of different physical systems including semiconductor heterostructures, graphene, quantum wires, quantum dots, and optical cavities (see [22] and references therein). We first introduce, in Section 2, the elements of the electrical phenomenon known as Coulomb drag, together with other possible theoretical frameworks compatible with the observed behavior. Then, in Section 3 we describe drag transverse voltage effects observed in a Nb/Al_1−x_O_x_/Pd_0.84_Ni_0.16_ (S/I/F) trilayer. The measurements have been performed at the liquid helium temperature (T = 4.2 K) by current biasing the superconducting strip and measuring the voltage induced on the unbiased ferromagnetic strip. An external magnetic field has also been applied perpendicularly to the plane of the trilayer. The data are compared with those obtained on Nb/Pd_0.84_Ni_0.16_ (S/F) and Nb/Al (S/N) bilayers, with the same geometry but in the absence of an insulating barrier. In Section 4, the results are qualitatively interpreted in terms of drag effect and peculiar interaction phenomena between the two layers.

## 2. Theory

In a typical experiment involving transport measurements for testing the behavior of a single unperturbed system, such as a linear conductor, when a current *I* is driven through the conductor, a voltage drop *V* is measured along the conductor itself, resulting in the Ohmic resistance R=VI. The interactions between charge carriers only cause corrections to the temperature dependence of transport coefficients and then to *R*. In a good metal, at low temperatures, *R* is mostly determined by disorder. The Drude model can be used to account for this behavior and provides an estimate of the magnitude of the resistance [23].

For a system made of two isolated conductors, a long-range interaction can occur which is the so-called Coulomb drag. It is due to a mutual friction that has been described [1,2,3] in terms of scattering between charge carriers belonging to the different layers. These scattering events are accompanied by energy and momentum transfer from the carriers in the active layer to the carriers in the passive layer. In this picture, if $I_{active}$ is the current flowing in one layer, such scattering events drag the carriers along the other, unbiased, passive layer, causing a voltage drop, $V_{passive}$. The friction can thus be measured through a transresistance defined as RD=VpassiveIactive. Semiconductor-based two-dimensional electron gases electrically separated have been used in experiments with varying amounts of Coulomb and tunnel coupling between the layers. Both Coulomb and tunneling effects can in fact contribute to *V_passive_*. In a structure made of two conducting layers separated by an insulating barrier, there is indeed a finite probability that electrons go through the barrier by means of a tunneling effect.

Coulomb effects can on the other hand, even in the absence of tunneling, produce new correlated states that can be detected through frictional drag. In fact, at low temperature, an intriguing scenario has been suggested [24,25] giving evidence for spontaneous interlayer phase coherence in a bilayer. Electron–electron interactions, within each layer as well as between the two layers, could in fact contribute to a transition into a new phase of quantum electronic matter. Into this new phase, electrons belong to both layers at the same time with a finite probability to stay in one or the other one. This uncertainty is not due to the presence of an interlayer tunneling but spontaneously develops as the system reaches the new state. This is a quantum state, and the Drude model cannot account anymore for describing it. The first experimental indication for unusual transport properties in bilayers came from Coulomb drag experiments performed in simply connected square geometry [26], with a current driven through one of the layers, the active layer, and voltage drops measured in the other one, the passive layer. At zero magnetic field, the drag resistance *R_D_* reflects momentum transfer due to interlayer electron–electron interactions [2,4]. When a magnetic field is applied, *R_D_* will be modified and a transverse resistance can appear when strong interlayer electronic correlations develop [5]. This transresistance depends on the interaction between the layers, as well as on the microscopic nature of each layer. In this quanto-mechanical picture, the transresistance is expected to be very small for low magnetic fields (fraction of mΩ in our case). A threshold current can arise, above which a transresistance can be detected even in the absence of contact between the layers [25]. Novel states of matters are extensively studied and classified in literature. Among these, a new type of interacting crystalline topological state, the topological mirror excitonic insulator, characterized by a quantized bulk charge polarization, has been investigated by means of field theory to incorporate the interaction effects in one dimension [27]. It has been reported that in excitonic insulators the presence of a static electric field in the dc transport regime can induce a charge pumping [28]. Exciton order induced in bilayer quantum spin Hall insulator by means of magnetic field has also been reported [29]. Hybrid structures containing superconductors, ferromagnets, and topological insulators have been modeled in order to investigate the induced voltage resulting from the magnetization dynamics [30]. Moreover, a study of the influence of the proximity effect in SIFS junctions on the density of states in the vicinity of the tunnel barrier and on the resistive branch of current–voltage characteristics has also been reported [31]. A cross-talk effect in a normal metal-insulator-superconductor has been observed in the past, with a signal in the unbiased layer detected close to the superconducting transition [11]. Transverse drag between a superconductor and a ferromagnet has been observed more recently, in [20]. In the present work we use a conventional superconductor, namely Nb, with a T_c_ of 9.2K, for one layer and a conventional ferromagnet for the other layer, in order to look for an induced voltage in the ferromagnet when current biasing the superconducting Nb.

## 3. Experiments

### 3.1. Sample Fabrication

Nb/Al_1−x_O_x_/Pd_0.84_Ni_0.16_ trilayer, Nb/Pd_0.84_Ni_0.16,_ and Nb/Al bilayers were deposited by a three-source dc magnetron sputtering. The system used in this work (Kenosistec, Milano, Italy), equipped with a load-lock chamber, operates at a base pressure of about 4 × 10^−8^ mbar, while the depositions are performed in Ar pressure of the order of 3–8 × 10^−3^ mbar, depending on the sputtered material, according to the following fabrication steps. A 100 µm wide strip geometry was first defined on a Si (100) substrate, then a 73 nm thick Nb layer was deposited at a rate of r_Nb_ = 0.3 nm/s. The Nb bridge was then obtained by lift off. Another 100 µm wide bridge was defined as before but in a cross geometry and the Al layer was sputtered on it in two steps at a rate of r_Al_ = 0.06 nm/s. First, a 1.7 nm thick layer was sputtered and exposed to air for 30 min allowing the formation of the oxide barrier. This first oxidation was followed by a second deposition of Al, this time for a 2.8 nm layer. The second oxidation was again performed in air, with a resulting estimated AlOx barrier thickness of 4.5 nm. The resulting bilayer was inserted in the deposition chamber and a Pd_0.84_Ni_0.16_ (=PdNi) film, 50 nm thick, deposited on it, at a rate of r_PdNi_ = 0.2 nm/s. Finally, the counter electrode was defined by a lift-off procedure. In order to rule out the role played by the oxide barrier on one hand, and by the ferromagnetic material on the other, S/F Nb/PdNi and S/N Nb/Al bilayers were fabricated, respectively, with an analogous procedure. All the layers were grown at room temperature and the deposition rates were monitored by a quartz crystal monitor calibrated by low-angle reflectivity measurements. The cross geometry of the samples, with overlap area 10 µm × 10 µm, was in this way obtained. All the deposited samples, their names and structures, are summarized in Table 1. The Pd and the Ni content in Pd_0.84_Ni_0.16_ alloy have been checked by Energy Dispersive Spectroscopy (Leo EVO 50, Karl Zeiss, Germany). For the Ni percentage present in our alloy, the ferromagnetic ordering at T = 4.2 K is well established, as reported in detail elsewhere [32].

The samples were electrically characterized by using a four-probe configuration, connecting the superconducting strip to a current supply Source Meter (Keithley mod. 2400, Cleveland, OH, USA) and the counter electrode to a Nanovoltmeter (Keithley mod. 2182A, Cleveland, OH, USA). An external magnetic field up to 2 T was applied perpendicularly to the plane of the heterostructures by means of a superconducting coil. A standard cryostat (American Magnetics, Inc. (AMI), Oak Ridge, TN, USA) was used for all the low temperature measurements.

### 3.2. Results

**Figure 2:**
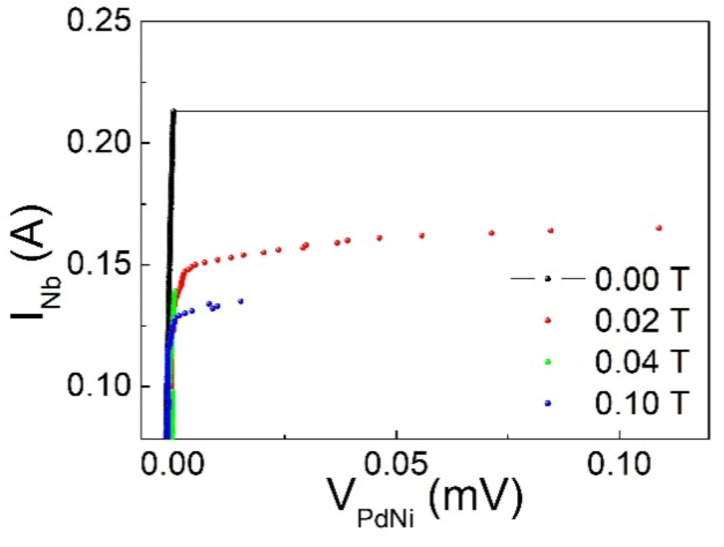
I–V curves at T = 4.2 K at different applied magnetic fields for a Nb/Al_1−x_O_x_/Pd_0.84_Ni_0.16_ trilayer in the case of current sent in Nb.

**Figure 3:**
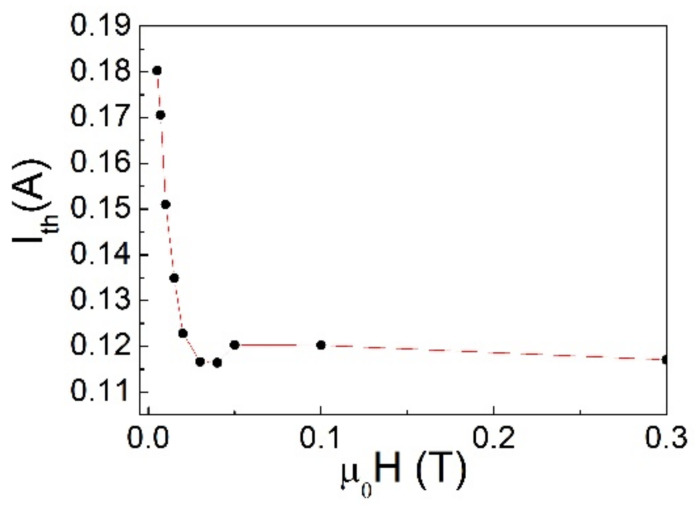
Behavior of I_th_ at different applied magnetic fields in a. Nb/Al_1−x_O_x_/Pd_0.84_Ni_0.16_ trilayer, when biasing the sample in the Nb layer (the dashed line is a visual guide).

**Figure 4:**
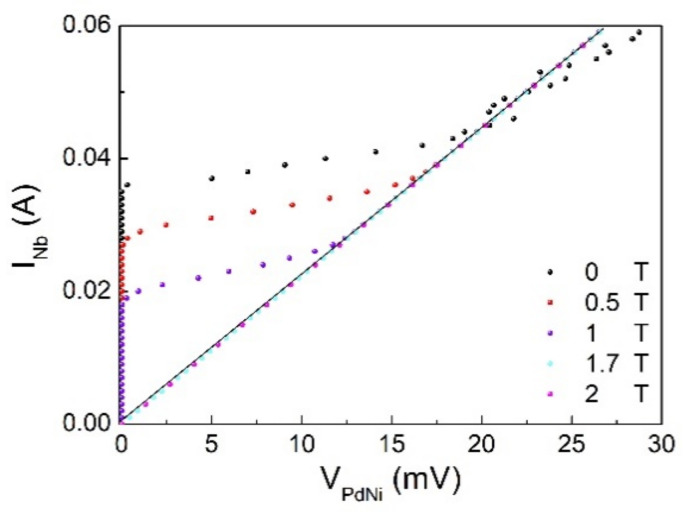
I–V curves at T = 4.2 K at different applied magnetic fields for a Nb/Pd_0.84_Ni_0.16_ bilayer with current sent in Nb.

**Figure 5:**
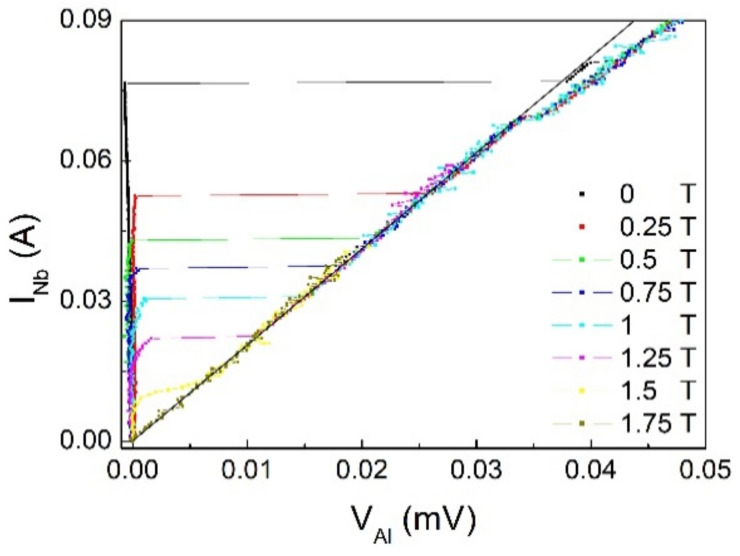
I–V curves at T = 4.2 K at different value of the magnetic fields for a Nb/Al bilayer with current sent in Nb.

Typical I–V curves of the S/I/F system at T = 4.2 K are shown in Figure 2 obtained with current flowing in Nb and voltage drop measured on F. It is evident that a non-zero voltage state appears in the unbiased F layer only when the bias current in the S layer overcomes a threshold current I_th_. The value of I_th_ is determined as the value of the current flowing in the S layer that causes a voltage drop of 0.1 mV in the F layer. It depends on the applied magnetic field. As shown in Figure 3, I_th_ strongly reduces, up to the magnetic field of about µ_0_H = 400 G (which is close to the value of the coercive field of the Pd_0.84_Ni_0.16_ strip [32] and much lower on the upper critical field of Nb). The high value of I_th_ (see also comments below to Figure 4 and Figure 5) and mainly its dependence on the external magnetic field rule out the possibility that we are measuring the superconducting critical current of the Nb film. For larger fields, up to almost µ_0_H = 2 T, I_th_ remains substantially constant (these data are not shown in the Figure 3).

A different behavior is observed for layers not electrically insulated. As shown in Figure 4 in the case of S/F bilayers, the current–voltage (I–V) behavior is similar to what is expected for the current–voltage characteristic of a superconductor [33,34,35,36]. In fact, since the Nb is in direct contact with Pd_0.84_Ni_0.16_, the I–V curve will resemble the behavior of Nb, including the proximity effect between S and F. In our data, a transverse, normal state resistance R_T_ = 0.45 Ω is measured, above the maximum zero-voltage current of about 0.04 A measured at zero field. This value, which can be interpreted in terms of the critical current of the Nb proximized to the ferromagnet, decreases by increasing the magnetic field, as expected for the I_c_ of a superconductor. Figure 5 shows the I–V curves measured at T = 4.2 K on the S/N bilayer with current flowing in S. In this case, the maximum value of current at zero voltage is higher consistent with a different degree of proximity effect between S and N. Compared with the S/F case, the transverse, normal state resistance is lower, R_T_~5 × 10^−4^ Ω.

**Figure 6:**
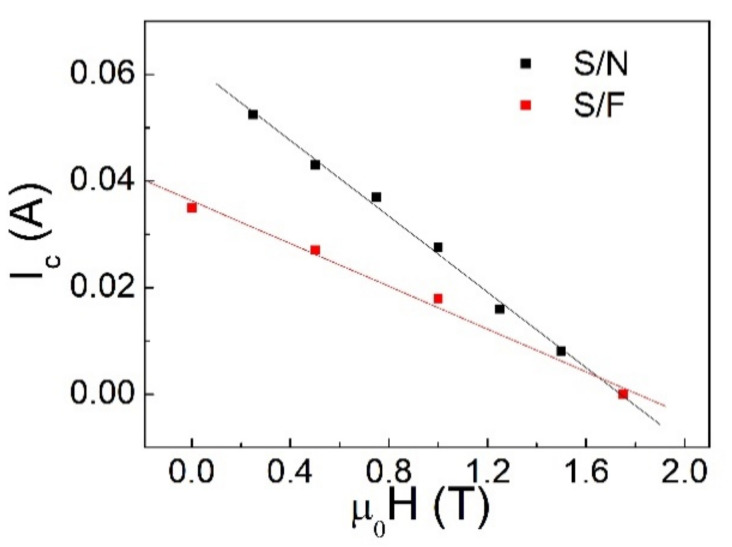
Dependence of I_c_ on the external applied field for Nb/Pd_0.84_Ni_0.16_ and Nb/Al bilayers (the lines are a visual guide).

In Figure 6, a comparison is shown between the critical measured in S/F and S/N bilayers.

Concerning the error bars not visible in the plots, it is required to specify that they are all so low due the high resolution of the instruments used that are not representable on the scales used for the plots (nanovolts for voltages, milliamps for currents, and millitesla for the magnetic fields).

## 4. Comments and Conclusions

The main feature emerging from our measurements in S/I/F structures is the presence of a threshold current, i.e., the value of the current in the superconducting strip above which an appreciable non-zero value of the voltage appears on the unbiased ferromagnetic strip. When the current overcomes the threshold value, the quasiparticle component in the superconductor is enhanced due to pair breaking. The induced measured voltage perpendicular to the bias current requires an additional mechanism for a charge/spin imbalance similar to an anomalous Hall effect caused by the spin polarized current in superconductors [37].

The application of a magnetic field perpendicularly to the plane of the samples influences the behavior of the threshold current: when the superconductor is current biased, this threshold reduces by raising the field, up to a value of H which is close to the coercive field of the ferromagnet (H_C_~400 G for Pd_0.84_Ni_0.16_) [27]. Then, the current threshold remains constant up to higher fields (µ_0_H = 2 T), see Figure 3.

A drag transresistance *R_D_* has been measured over I_th_. More investigation is needed in order to understand and properly describe the experimental data.

As already observed in a similar system measured in the same cross configuration [20], the S/I/F data can be explained in terms of a drag voltage induced by the Coulomb interaction between the Fermi electron gases of the two separated layers. Other different and non-trivial effects may compete in these systems, such as, for instance, tunneling or other contributions involved in the interaction [20]. These can be related to the degree of contact between the two subsystems as well as to the nature of the non-superconducting electrode. Due to spin effects, mesoscopic local and non-local thermopower effects could also be invoked, as those investigated in S/F heterojunctions [38].

The situation is different when no insulating barrier between the layers is present in the S/F as well as in the S/N system. In this case, the maximum zero voltage current can be explained in terms of the critical current of S and varies monotonically with the external field, as represented in Figure 6. This confirms the role played by the barrier, which uncouple the two strips, making the system behave in a completely different way with respect to the case in which it is absent.

In conclusion, we have proposed a possible experiment for observing drag voltage effects in a S/I/F system. A threshold current, sensitive to the applied magnetic field, has been observed. We speculate that the barrier in S/I/F plays an important role since the results are different in the absence of the insulator between the S and F (or N) layers.

## Figures and Tables

**Table 1:** Characteristics of the samples.

	Substrate	d_Nb_(nm)	d_Al_(nm)	d_AlOx_(nm)	d_PdNi_(nm)
Nb/Al_1−x_O_x_/Pd_0.84_Ni_0.16_	Si	73	-	4.5	50
Nb/Pd_0.84_Ni_0.16_	glass	100	-	-	100
Nb/Al	Si	73	50	-	-

## Data Availability

The data presented in this study are available on request from the corresponding author.
